# Gender Affects Skin Wound Healing in Plasminogen Deficient Mice

**DOI:** 10.1371/journal.pone.0059942

**Published:** 2013-03-20

**Authors:** Birgitte Rønø, Lars Henning Engelholm, Leif Røge Lund, Andreas Hald

**Affiliations:** 1 Center for Molecular Pathology, Department of Laboratory Medicine, Lund University, Malmo, Sweden; 2 Department of Cellular and Molecular Medicine, Faculty of Health Sciences, University of Copenhagen, Copenhagen, Denmark; 3 Finsen Laboratory, Rigshospitalet, Copenhagen, Denmark; 4 Biotech Research and Innovation Centre, University of Copenhagen, Copenhagen, Denmark; University of Iowa, United States of America

## Abstract

The fibrinolytic activity of plasmin plays a fundamental role in resolution of blood clots and clearance of extravascular deposited fibrin in damaged tissues. These vital functions of plasmin are exploited by malignant cells to accelerate tumor growth and facilitate metastases. Mice lacking functional plasmin thus display decreased tumor growth in a variety of cancer models. Interestingly, this role of plasmin has, in regard to skin cancer, been shown to be restricted to male mice. It remains to be clarified whether gender also affects other phenotypic characteristics of plasmin deficiency or if this gender effect is restricted to skin cancer. To investigate this, we tested the effect of gender on plasmin dependent immune cell migration, accumulation of hepatic fibrin depositions, skin composition, and skin wound healing. Gender did not affect immune cell migration or hepatic fibrin accumulation in neither wildtype nor plasmin deficient mice, and the existing differences in skin composition between males and females were unaffected by plasmin deficiency. In contrast, gender had a marked effect on the ability of plasmin deficient mice to heal skin wounds, which was seen as an accelerated wound closure in female versus male plasmin deficient mice. Further studies showed that this gender effect could not be reversed by ovariectomy, suggesting that female sex-hormones did not mediate the accelerated skin wound healing in plasmin deficient female mice. Histological examination of healed wounds revealed larger amounts of fibrotic scars in the provisional matrix of plasmin deficient male mice compared to female mice. These fibrotic scars correlated to an obstruction of cell infiltration of the granulation tissue, which is a prerequisite for wound healing. In conclusion, the presented data show that the gender dependent effect of plasmin deficiency is tissue specific and may be secondary to already established differences between genders, such as skin thickness and composition.

## Introduction

The fibrinolytic activity of plasmin, the active form of the zymogen, plasminogen (Plg), is important for mucosal membrane maintenance and organ function. In humans, loss of plasmin dependent fibrinolysis leads to abnormal wound healing and development of pseudo membranes on mucosal tissues throughout the body. The gastrointestinal tract, respiratory system, and genitourinary tract are often strongly affected, which may ultimately lead to organ failure and increased morbidity. However, the most noticeable clinical syndrome in these patients is ligneous conjunctivitis represented by fibrin rich lesions on the eyelids, which may be resolved by local treatment with Plg [1], [2]. Similarly, mice that harbor deficient Plg alleles (Plg^−/−^) develop normally into adulthood but will eventually suffer from inflammatory lesions and ulcers throughout the gastrointestinal tract, lungs, and reproductive organs [3]. The progressive deformation of these organs results in organ dysfunction and wasting of the animals, which rarely live for more than 30 weeks. In addition to these syndromes, fibrin depositions, which are associated with infiltration of inflammatory cells, accumulate in the liver throughout the lifespan of Plg^−/−^ mice [3], [4]. Moreover, wound healing in humans with plasmin deficiency and in Plg^−/−^ mice is severely retarded and associated with accumulation of fibrin in the provisional matrix [5], [6]. Studies in mice have also shown that the initial recruitment of immune cells to an inflamed tissue is dependent on Plg [7], though the role of fibrin in this setting is not established and may vary according to choice of model. During the past decade, plasmin has been shown capable of activating matrix tethered growth factors and other extracellular proteases [8], [9]. These functions may be important during the process of tissue reconstruction following chemical or physical damage as a result of e.g. chemotherapy [10]. During such processes, a large number of extracellular proteases are actively present, and some of these share, besides their expression pattern, also substrates [11], [12]. Thus the specific contribution of individual proteases in the intricate system of extracellular proteolysis during a biological event is a challenge to identify [13], [14].

Besides the important functions of plasmin in maintaining homeostasis and tissue integrity, plasmin and the Plg activation (PA) system also mediate pathological processes, such as tumor growth and cancer metastasis [15]. The mechanism linking the PA-system to these malignant events varies according to the tissue of origin and to the metastatic site [15], [16]. Depending on these factors, the function of the PA system has been shown to involve clearance of extracellular matrix, release/activation of tumor growth factors, and securing the patency of the tumor vasculature [15], [17]. Recently, it was demonstrated that skin tumor growth in mice was retarded by the absence of plasmin driven fibrinolysis, which serves to clear tumor thrombosis and maintain a sufficient blood supply to the growing tumors [18]. Surprisingly, this effect of plasmin was restricted to male mice and thereby suggesting that Plg^−/−^ female mice are not prone to the same decree of tumor thrombosis or can resolve fibrin clots by expressing fibrinolytic protease(s) besides plasmin. Alternatively, the endogenous differences in skin composition between genders could be responsible for the different effects of plasmin deficiency on skin tumor growth in male and female mice.

We here describe a systematic investigation of the phenotype of Plg deficiency in male and female mice, and reveal an effect of gender that is restricted to the skin. This gender effect could not be resolved by ovariectomy (OVX) thus suggesting that it is likely linked to variations in skin composition and less likely to a hormone dependent expression of a fibrinolytic protease.

## Materials and Methods

### Ethics Statement

All experimental animals were monitored daily by trained animal caretakers. Animals showing signs of distress were euthanized by cervical dislocation. All experiments were conducted according to institutional and national guidelines. The experiments performed in the current project were specifically approved by the Danish Animal Experiments Inspectorate (permit number 2010-561-1778). Following the experiments, animals were euthanized by anaesthetizing using hypnorm/dormicum (fentanyl 0.1 mg/ml, fluanison 5 mg/ml, midazolam 5 mg/ml) followed by perfusion, as described below.

### Animal Breeding

Both FVB/n and C57Bl/6 mice were used for wound healing studies. Tissue libraries were derived from FVB/n mice [4]. The thioglycollate (TG) induced peritonitis experiments were performed in C57Bl/6 mice. The FVB/n Plg^−/−^ mice and their littermate controls were generated by breeding heterozygous mice (Plg^−/+^), that had been backcrossed into an FVB/n background for at least 30 generations (Panum Institute, Copenhagen, Denmark). C57Bl/6 Plg^−/+^ mice were likewise backcrossed for at least 30 generations into the C57Bl/6 background. All mice were bred in the SPF facility at University of Copenhagen and during experimentation they were housed individually. Genotyping was performed as previously described [18] and following the experiments all used mice were regenotyped.

### Ovariectomy

Mice of approximately five weeks of age were anaesthetized using hypnorm/dormicum and the ovaries were excised as previously described [18]. Briefly, the ventral skin and muscle tissue were cut with scissors and the ovaries resected and the wound suturated. Similar sham surgeries were performed in both male and female mice. One week following OVX, incisional wounding was performed as described below. At the termination of the experiment, successful OVX was confirmed by isolating and weighing the uteri.

### Incisional Wounds

Six weeks old mice were used for incisional wound healing studies as described previously [4]. Briefly, mice were anaesthetized with hypnorm/dormicum and a 20 mm long full skin thickness incision was made along the dorsal midline. The mice were housed individually and wound lengths were measured every other day until healed, defined as complete wound closure and full restoration of the epidermis (termed reepithelialization). At this point, the mice were euthanized with hypnorm/dormicum and perfused with 10 ml phosphate buffered saline (PBS) followed by 10 ml of a 4% paraformaldehyde solution (PFA). Next, the wounded skin areas were isolated with scissors and the tissues were incubated overnight in 4% PFA and then transferred to 70% ethanol and kept at 4°C until further processed and embedded in paraffin.

### Thioglycollate Induced Peritonitis

Six weeks old mice were injected intraperitoneally (IP) with 1 ml of a 3.85% TG brew (Difco, BD, Denmark, Cat.# 243210), which had been incubated for at least four weeks at 4°C. After three days, the mice were anaesthetized with hypnorm/dormicum, the abdominal skin removed leaving behind the abdominal muscle tissue, and the mice were then injected IP with 4 ml PBS and gently shaken on a lab vibrator for 1 min. The lavage fluid was harvested and the cell concentration was defined using an automated cell counter (NucleoCounter NC-100, ChemoMetec, Allerød, Denmark).

### Liver and Skin Samples

Liver and skin samples from naïve wildtype (Wt) and Plg^−/−^ mice were derived from a tissue collection containing samples isolated from mice with an age of eight, 12 and 26 weeks [4].

### Histology

Paraffin embedded tissues were sectioned, rehydrated and stained using a standard H&E staining protocol. For immunohistochemical detection of fibrin(ogen) and CD34, the following antibodies were used: rabbit-anti-mouse fibrin(ogen) [19] diluted 1∶2000 and rat-anti CD34 (HyCult Biotech, Uden, the Netherlands, HM1015-3915M12) diluted 1∶100, followed by incubation with a rabbit-anti-rat antibody (Dako, Glostrup, Denmark, K, E468) diluted 1∶100. Chromogen staining was achieved using the EnVision^+^ system (Dako, Glostrup, Denmark, K4003) in combination with NovaRED HRP substrate (VWR international, Herlev, Denmark, SK-4800). Stained sections were scanned using a motorized Olympus BX51 microscope with a 20X objective controlled by Visiopharm software (Visiopharm, Hørsholm, Denmark) or by a NanoZoomer-2.0HT (Hamamatsu, Denmark) using a 20X or 40X objective. In wound tissue, cell counting was performed in randomly selected areas either containing or devoid of fibrin. The average size of these regions of interest (ROIs) was 0.035 mm^2^. The average thickness of the epidermis was derived from the area of the hyperplastic epidermis divided by the length. The combined area of fibrin rich lesions in the provisional matrix was determined using the staining analysis software VisiomorphDP, which is part of the Visiopharm software package (Visiopharm, Hørsholm, Denmark). In the liver, the degree of fibrin deposition was determined on one whole scanned tissue slide from each liver using VisiomorphDP (Visiopharm, Hørsholm, Denmark). Likewise, was the area of CD34 positive staining quantitated by use of VisiomorphDP on whole tissue slides scanned at 40X.

### Statistical Analyses

Statistical analyses were performed using Two-way ANOVA, One-way ANOVA with a Newman-Keuls post test or with student’s t-tests.

## Results

### Fibrin Accumulation in the Liver of Naïve Mice is Unaffected by Gender

**Figure 1:**
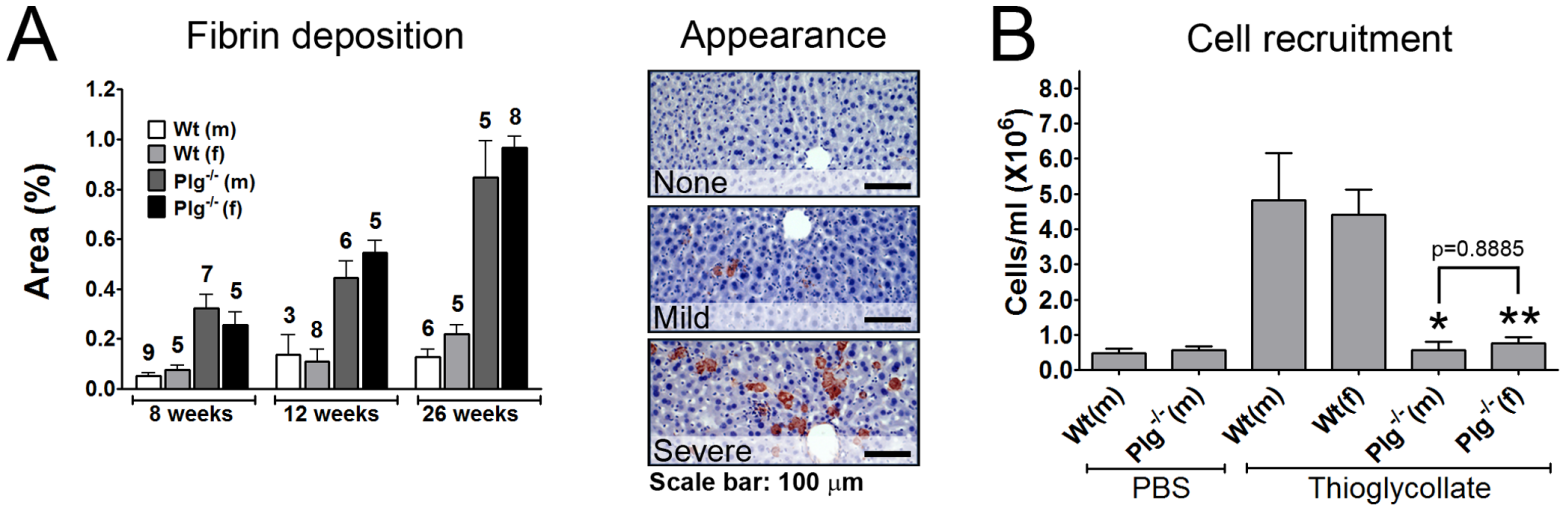
Deposition of fibrin in the liver and plasminogen dependent cell migration is unaffected by gender. The effect of gender on spontaneous fibrin deposition in the liver and recruitment of inflammatory cells was tested in Plg^−/−^ mice. (A) The degree of fibrin deposition in the liver in Plg^−/−^ mice and Wt controls of both genders was determined by computerized quantitative immunohistochemistry. The number of mice in each group is shown above each column. The difference between Wt and Plg^−/−^ mice was significantly different at all time points (p<0.01), while there was no difference between genders. Representative liver stainings from mice with varying degrees of liver fibrin depositions are shown on the right. (B) The effect of Plg deficiency on Plg dependent inflammatory cell recruitment was tested by IP injections of TG and determining the concentration of cells in the peritoneal lavage fluid after three days. *, p<0.05; **, p<0.01 compared to TG treated Wt male and female mice. Data were analyzed with a one-way ANOVA and a Newman-Keuls post test. n = 3–8 in each group.

Mice lacking functional Plg display large and disseminated fibrin rich lesions in the liver [3]. This pathological condition is also observed in mice with a deficient PA system [20] or in mice treated with PA inhibitors [21], [22]. In order to investigate if gender affects the accumulation of fibrin rich lesions in unchallenged Plg^−/−^ mice, we conducted a histological analysis of livers derived from a cohort of Plg^−/−^ mice. Immunohistochemical staining revealed no signs of fibrin rich lesions in livers from Wt mice regardless of age (up to 26 weeks). However, in Plg^−/−^ mice, fibrin rich lesions were evident already at an age of eight weeks. Computerized staining analysis revealed that the number, size and combined area of these lesions increased significantly up to the age of 26 weeks. However, when comparing livers from male and female Plg^−/−^ mice it was evident that there were no differences between genders (Figure 1A). Together with previous reports stating that the plasma fibrinogen levels are constant between genders [18], these observations provide evidence that naïve Plg^−/−^ female mice are just as incapable of clearing deposited fibrin in the liver as are Plg^−/−^ male mice.

### Both Male and Female Mice Depend on Plasminogen in the Acute Recruitment of Inflammatory Cells

The recruitment of inflammatory cells to the peritoneal cavity following an IP injection of a proinflammatory agent such as thioglycollate (TG) is known to depend on Plg [7]. Likewise has an obstructed PA system been shown to ameliorate the inflammatory response during chemically induced peritonitis [23]. This role of Plg has been ascribed to plasmin dependent matrix metalloproteinase (MMP)-9 activation and subsequent basement membrane degradation, which in turn allows inflammatory cells to migrate to the peritoneal cavity [8]. However, the possibility of a role for direct plasmin driven fibrinolysis in this process has yet to be addressed.

To test if the obstructed recruitment of inflammatory cells to the peritoneal cavity due to lack of Plg is gender dependent, we compared the response to IP injected TG in Wt and Plg^−/−^ mice (C57Bl/6 backcrossed for at least 30 generations) of both genders totaling 26 mice. Three days after TG injection, Wt mice presented an almost tenfold increase in the concentration of cells in the peritoneal cavity. This response to TG was independent of gender. When comparing the response to TG in Wt and Plg^−/−^ mice, a striking difference was observed, as the latter mice seemed completely unaffected by the administration of TG. This, however, was regardless of gender (p = 0.8885 for the difference between genders) (Figure 1B). The lack of a gender effect on the acute Plg-dependent recruitment of inflammatory cells does not support the notion that female mice may circumvent the lack of Plg by expressing other protease(s) with a similar repertoire of substrates.

### Dysfunctional Skin wound Healing in Plg^−/−^ Mice is Modulated by Gender

**Figure 2:**
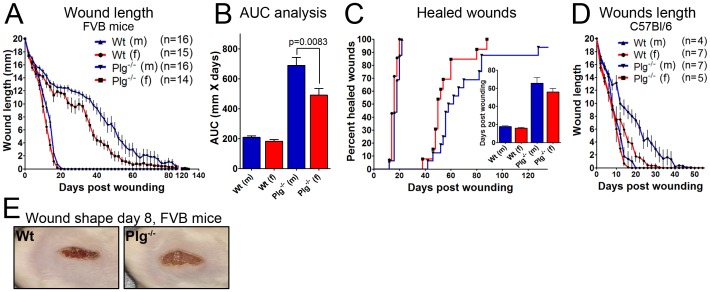
Skin wound healing in plasminogen deficient mice is affected by gender. Skin wound healing was tested by measuring time to complete reepithelialization of a 20 mm long full skin thickness incisional wound on the back. (A) The wound length in FVB mice was determined every other day using digital calipers. A substantial difference was observed between male and female Plg^−/−^ mice (p<0.0001). (B) The area under the curve analysis revealed a significant difference between genders in the Plg^−/−^ mice. (C) The fraction of fully reepithelialized wounds is presented as a function of time. The bar graph shows the average time to complete reepithelialization. (D) A wound healing study identical to the one carried out in FVB mice were performed in C57Bl/6 mice. Similar results were obtained in C57Bl/6 mice with a significant difference between wound healing times in male and female Plg^−/−^ mice (p = 0.01). (E) Representative pictures of skin wounds in Wt and Plg^−/−^ male mice eight days after the incision.

It has previously been demonstrated that Plg deficiency affects chemically induced skin tumor growth differently in male and female mice. Whereas Plg deficiency decreased tumor burden by 52% in male mice, biallelic Plg disruption did not affect the skin tumor burden in female mice compared to littermate control mice [18]. However, Plg deficiency has on other occasions been shown to affect biological events in female mice, involving substantial tissue remodeling, such as mammary gland involution [24], [25]. Based on these reports and on the lack of gender effects on fibrin accumulation in the liver and on recruitment of inflammatory cells in Plg^−/−^ mice, we speculated that gender only affected the phenotype of Plg^−/−^ mice in terms of skin pathologies. As it has been thoroughly documented that skin wound healing is highly dependent on plasmin driven fibrinolysis [26], we compared the capacity of Wt and Plg^−/−^ FVB mice of both genders to heal 20 mm long incisional skin wounds on the back. No difference in the time to complete skin wound healing was observed between Wt male and female mice (17.7±0.66 and 16±0.62 days for males and females, respectively), while the time needed to complete wound healing in Plg^−/−^ mice of both genders was markedly delayed compared to the Wt mice (Figure 2A–C,E). However, we also noted a distinct difference in wound healing between Plg^−/−^ male and female mice. Already five days post wounding, Plg^−/−^ female mice revealed faster wound healing than Plg^−/−^ male mice. This trend was further emphasized from 20 days post wounding. Interestingly, as the wounds in the male and female Plg^−/−^ mice were approximately 95% healed (75 days post wounding) no differences between the genders could be seen (Figure 2A–C). The healing time curves from Plg^−/−^ male and Plg^−/−^ female mice were significantly different (p<0.0001), while there were no differences in terms of average time to complete wound closure (p = 0.2023). These data suggested that it is not only skin tumor growth in Plg^−/−^ mice that is affected by gender but also other types of tissue remodelling processes that takes place in the skin.

To further substantiate the concept of a gender dependent effect of Plg deficiency in terms of wound healing, a wound healing study similar to the one performed in FVB mice was performed in fully backcrossed C57Bl/6 mice (backcrossed for at least 30 generations). This experiment was carried out by independent research technicians that were unaware of the gender dependent phenotype observed in FVB mice. This experiment confirmed the existence of a gender dependent phenotype in relation to skin wound healing in Plg^−/−^ mice. Indeed, the effect of gender was more pronounced in C57Bl/6 mice than in the corresponding FVB mice (Figure 2D). Noteworthy is that, in the applied wound healing model, both Plg^−/−^ as well as the Wt control mice on the C57Bl/6 background heal much faster than the corresponding FVB mice. Pronounced differences between the FVB and C57Bl/6 mice strains lacking Plg^−/−^ is also evident in regard to other phenotypic features, such as the development of rectal prolapses, which appear significantly faster in C57Bl/6 mice (unpublished results). Though C57Bl/6 mice with a dysfunctional PA-system also display fibrin depositions in the liver [22], a direct comparison of the liver pathology between Plg^−/−^ FVB and C57Bl/6 mice has never been performed. Thus, the phenotype of Plg deficiency may very well be partly strain dependent.

### Skin Thickness and Composition Differs between Genders but are Unaffected by Plasminogen Deficiency

**Figure 3:**
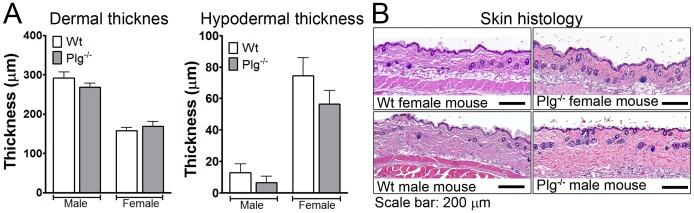
Plasminogen deficiency does not affect the structure of naïve skin. The effect of Plg deficiency on the known differences in skin histology between genders was investigated using back skin from naïve Plg^−/−^ mice and Wt controls (8–12 weeks of age). n = 8–12 for each group. (A) The thickness of the dermis and hypodermis was determined by microscopy. (B) Representative skin sections from eight week old male and female mice.

The obtained data, suggesting that the ability of Plg^−/−^ mice to execute tissue remodelling processes in the skin is affected by gender, prompted us to investigate if the known differences between the genders in terms of thickness of the dermal and hypodermal compartments were affected by Plg deficiency. For this purpose we analyzed normal skin samples from the previous mentioned cohort tissue collection [4]. We found, as expected, that naïve Wt male mice had thicker dermis and thinner subdermis than Wt female mice. Further studies revealed that these differences were not affected by Plg deficiency (Figure 3). Thus a differential gender effect of Plg deficiency on skin composition is not likely responsible for the gender differences observed with regard to skin wound healing in Plg^−/−^ mice.

### The Subtle Effect of Plasminogen Deficiency in Female Mice is not Augmented by Ovariectomy

Expression of distinct extracellular matrix proteases with a potential fibrinolytic activity may to some extent be regulated by sex-hormones. It can thus not be ruled out that female mice, during the process of skin wound healing, have the capacity to express fibrinolytic proteases, which can compensate for the lack of plasmin. To test this hypothesis we analyzed the effect of OVX on skin wound healing in Wt and Plg^−/−^ mice.

**Figure 4:**
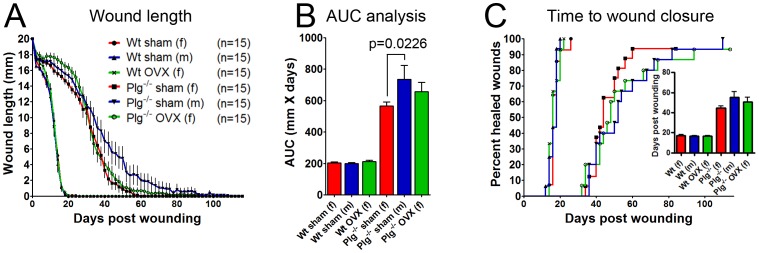
Ovariectomy does not induce a male like phenotype in plasminogen deficient female mice. A possible effect of ovarian derived hormones on skin wound healing in Plg^−/−^ mice was tested by excision of the ovaries before a 20 mm full thickness incisional skin wound was made. (A) Wound lengths were measured using digital calipers every other day until completely healed. No statistical significant difference was found between the different groups belonging to Wt mice. The difference between sham operated male and female Plg^−/−^ mice were statistically significant (p<0.05), while no significant difference was found between OVX operated Plg^−/−^ mice and the sham operated Plg^−/−^ mice. (B) An area under the curve analysis showed no significant effect of OVX in Plg^−/−^ mice, while the difference between genders was significant. (C) The percentage of mice with completely reepithelialized wounds presented as a function of time. The bar graph shows the average time to complete reepithelialization.

As previously observed, we found no significant differences between Wt male and female mice in terms of wound healing. Furthermore, in Wt mice, OVX did not significantly affect wound healing when compared to sham operated male and female mice. In Plg^−/−^ mice, the gender differences in wound healing were still clearly evident despite sham and OVX surgeries. However, in contrast to Wt mice, OVX in Plg^−/−^ mice led to a subtle delay in the initial phase of the wound healing process compared to both sham Plg^−/−^ male and female mice. However, around day 30 post wounding, it was no longer possible to distinguish between sham and OVX Plg^−/−^ female mice (Figure 4A,B), resulting in similar overall time to complete wound healing in the two groups (Figure 4C). Thus female sex-hormones may contribute in orchestrating the initial phase of wound healing, as suggested by others [27], but they do not seem to be responsible for the overall difference in wound healing between male and female Plg^−/−^ mice.

### Overt Differences between Genders in Epithelial Structure and Cell Invasion during Wound Healing

**Figure 5:**
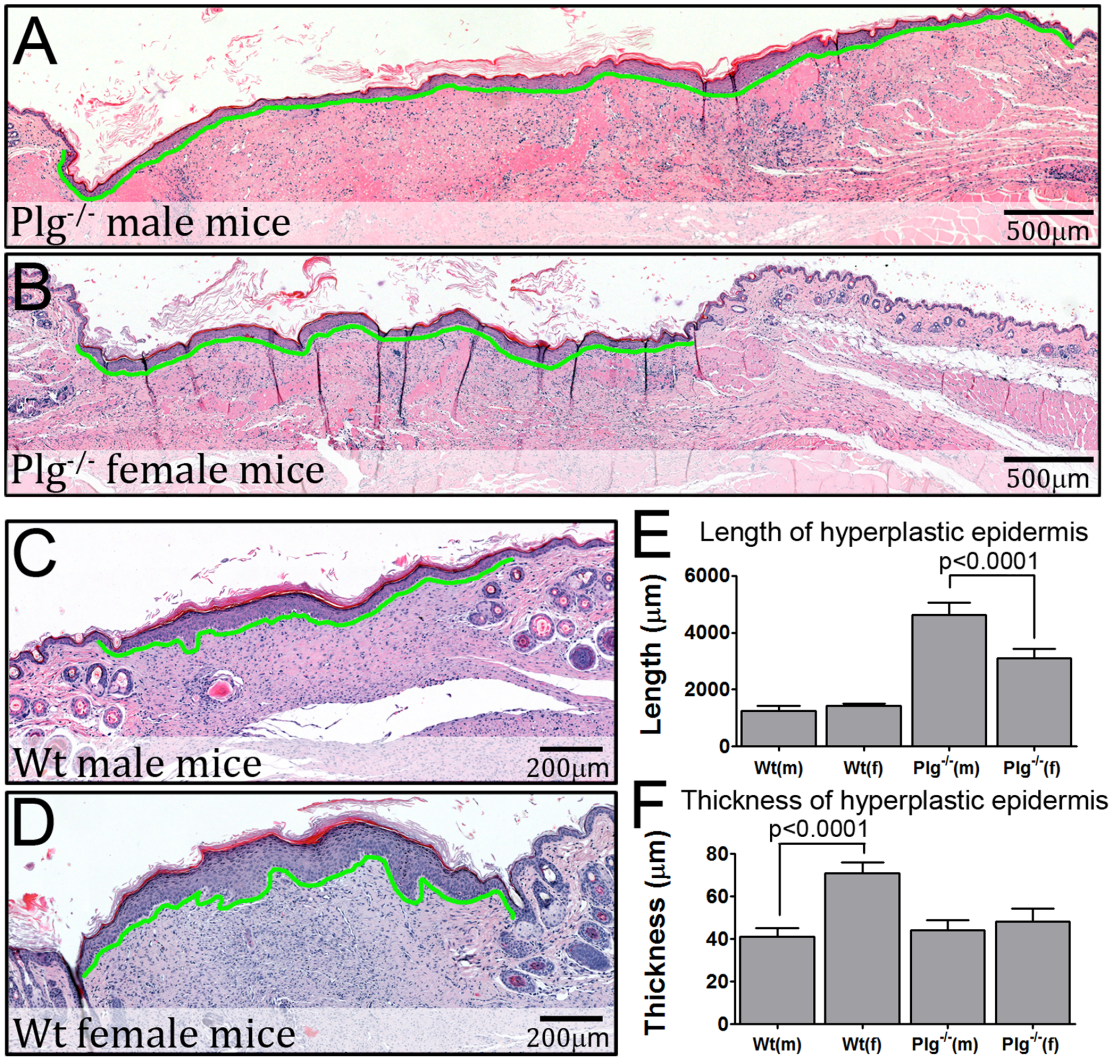
The length of the hyperplastic epidermis and dermal wound gap is affected by gender. Histological analysis of H&E stained sections from fully reepithelialized wounds. (A–D) representative sections of healed wounds from Wt and Plg^−/−^ mice of both genders. The hyperplastic epidermis is underlined by green. (E & F) The length and average thickness of the hyperplastic epidermis. n = 9–11 for each group.

To further elucidate the impact of genders on wound healing in Plg^−/−^ mice, we performed a histological analysis of reepithelialized wounds (skin biopsies were harvested within 24 hours of wound closure). The wounds all displayed a hyperplastic epidermis, which covered a dermal wound gap containing yet unresolved granulation tissue. The lengths of the hyperplastic epidermis and wound gap were similar in Wt male and female mice, while the thickness of the hyperplastic epidermis was increased in Wt females. Interestingly, the lengths of the hyperplastic epidermis and dermal wound gap were significantly longer in Plg^−/−^ mice than in Wt mice, and furthermore, the lengths were 48% longer in Plg^−/−^ male than in Plg^−/−^ female mice (Figure 5A–F). These analyses show that, when wounds in Plg^−/−^ mice are fully reepithelialized, the underlying matrix is only to a limited extend reconstructed, as previously described [11]. In contrast, wounds in Wt mice have much less granulation tissue (dermal wound gap) upon reepitheliazation despite the fact that reepithelialization happens much faster in Wt mice than in Plg^−/−^ mice. Thus proliferation and migration of keratinocytes, which serves to reepithelialize wounds, seem less affected by Plg deficiency than does the rearrangement and contraction of the underlying granulation tissue.

**Figure 6:**
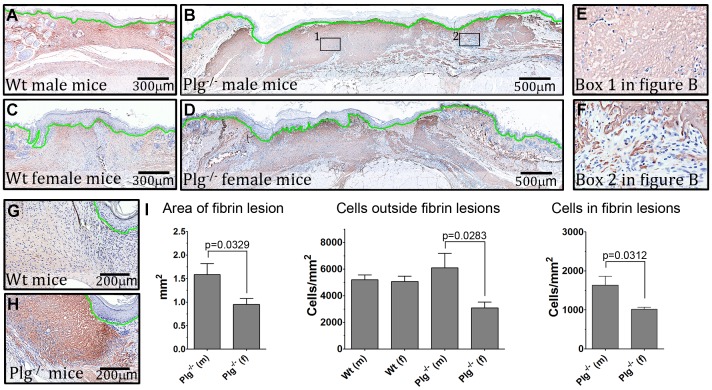
Decreased cell infiltration in fibrin rich areas of wound tissues. Fibrin(ogen) staining of skin wound sections. The epidermis is underlined by green, fibrin(ogen) is stained brown with NovaRED, and nuclei are stained blue with a hematoxylin counter stain. (A–D) representative stainings of wound tissues from Wt and Plg^−/−^ mice of both genders. (E & F) enlarged micrographs of areas boxed in B. (G & H) sections from 50% healed wounds in the back skin of male mice, which show the area between the advancing keratinocytes and the underlying muscle tissue Note the lack of cells in this region in the Plg^−/−^ mice. (I) quantifications of the total area of fibrin(ogen) positive lesions in the provisional matrix and quantifications of the number of cells within and outside this area. n = 9–11 for each group.

A quantitative histological assessment of fibrin deposition and cell concentration in the reepithelialized wounds revealed that the combined area staining positive for fibrin in the provisional matrix in Plg^−/−^ male mice was 62% higher than in Plg^−/−^ female mice (no noteworthy fibrin depositions were found in Wt mice of either gender). Furthermore, the concentration of cells in the fibrin rich areas was decreased by almost 80% in comparison with areas not containing fibrin. In addition, the granulation tissue in Plg^−/−^ female mice contained fewer cells than in Plg^−/−^ male mice, both within and outside the fibrin rich areas (Figure 6A–I). The observed difference in the area of fibrin rich lesions, and thus cell concentration, in male and female Plg^−/−^ mice could explain the gender dependent difference in wound healing time in Plg^−/−^ mice.

The notion that deposited fibrin blocks the recruitment of cells to the granulation tissue at an early time point was further supported by analyses of 50% reepithelialized wound tissues harvested from Plg^−/−^ mice, which revealed that the cellular infiltration of the granulation tissue with cells from the adjacent healthy skin was severely obstructed due to massive fibrin depositions (Figure 6G,H). This abolished recruitment of cells is most likely directly responsible for the lack of cells in the fibrin rich areas of the provisional matrix in the later fully reepithelialized wounds.

**Figure 7:**
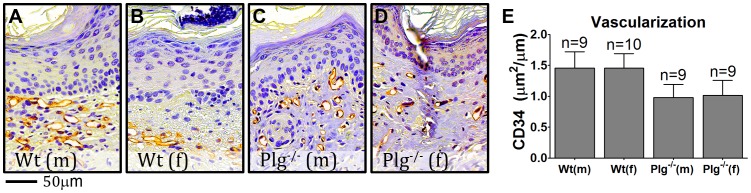
Gender does not affect the vessel concentration in fully reepithelialized wounds. CD34 stainings of fully reepithelialized wounds were performed to identify new vessels. (A–D) CD34 positive vessels were detected in the dermis proximal to the hyperplastic epidermis both in Wt and Plg^−/−^ mice of both genders. (E) Computerized staining analysis on whole wound sections reveal that Plg^−/−^ mice had a tendency to express less CD34 (area wise) than Wt mice while gender did not affect the level of CD34 staining.

Wound healing is intimately associated with the generation of a vascular network throughout the granulation tissue. This process depends on the interaction between endothelial and matrix proteins as well as proteolytic matrix reorginization by MMPs and the PA system [28]–[30]. To investigate if vessel formation in skin wounds in Wt and Plg^−/−^ mice were affected by gender, fully reepithelialized wounds stained for CD34 were scanned at 40X and subjected to computerized staining quantification. The combined area of CD34 positive vessels per µm wound tissue (defined by the presence of a hyperplastic epidermis) was compared. CD34 positive vessels were mainly observed within close distance to the epidermis in both Wt and Plg^−/−^ mice of both genders and no overt differences were observed in terms of size or shape between these groups of mice (figure 7A–D). The quantification of the CD34 staining signal revealed no differences between genders, while there was a tendency of Plg^−/−^ mice having decreased amounts of CD34 compared to Wt mice (Figure 7E).

## Discussion

In the present study the effect of gender on the phenotype of Plg deficiency was illuminated. Three well described characteristics were assessed; spontaneous accumulation of fibrin in the liver, Plg dependent cell recruitment to the peritoneal cavity, and incisional skin wound healing. Among these, only skin wound healing in Plg^−/−^ mice was affected by gender. Furthermore, the gender specific differences in naïve skin described in Wt mice were unaffected by Plg deficiency. Histological examination of healed wounds revealed that the combined area of fibrin rich lesions in the provisional matrix was larger in male Plg^−/−^ mice than in female Plg^−/−^ mice, and that these fibrin rich lesions contained far less cells than the surrounding wound matrix in both male and female mice. The difference in the area of fibrin rich lesions between genders correlated well to the differences in the length of the hyperplastic epidermis and amount of granulation tissue in healed wounds as well as to the measured difference in time to complete wound reepithelialization.

It is evident that the general phenotype of unchallenged Plg^−/−^ mice is the same in males and females, though subtle differences may be observed [4]. The recent discovery of a gender dependent effect of Plg deficiency on chemically induced skin tumor growth was therefore highly surprising [18]. The mechanism underlying this gender effect has not been fully elucidated, but may relate to decreased fibrin depositions in the skin and/or tumor thrombosis [18], both of which may affect skin tumor growth. Additionally, it was shown that male and female mice contained the same amount of plasma fibrinogen throughout their lifespan [18], thus ruling out such a difference as being responsible for the gender effects on the Plg^−/−^ phenotype. However, as spontaneous *in vivo* clot lysis, determined by intra-venous injections of radiolabeled fibrin clots, is remarkably reduced in Plg deficient mice compared to Wt mice [31], we cannot exclude that these differences may partly be accountable for the dissimilarities between the genders observed in Plg^−/−^ mice. Yet, we find this questionable as identical levels of fibrin depositions in the liver of male and female Plg^−/−^ mice were observed, which not only suggests that the livers in male and female Plg^−/−^ mice are equally degenerated over time, but also that a putative but insufficient extravascular fibrin clearance occurs at the same rate in both genders. Furthermore, the evident lack of a gender effect on the recruitment of immune cells to the peritoneal cavity in response to TG injections, suggests that males and females depend equally on plasmin activity to execute an inflammatory response. In accordance with these observations, Plg deficiency has not previously been reported to show a gender effect in macrophage recruitment [8], though experiments have been carried out in mice of both genders. Despite the hampered recruitment of immune cells in Plg^−/−^ mice challenged with a proinflammatory agent, inflammatory reactions seem to be fully executed in some chronic pathological settings, such as in the development of inflammatory colon lesions and skin tumors [4], [18], while ameliorated in other models [32].

In contrast to fibrin deposition in the liver and immune cell recruitment, we found that incisional skin wound healing in Plg^−/−^ mice was highly dependent on gender. The intricate process of wound healing involves fibrin clearance, which likely facilitates the recruitment of cells into the granulation tissue and/or keratinocyte migration [5], [26]. Besides increased activity of the PA-system during wound healing processes, zymographic studies on wound tissue have also revealed a transient increase in the activity of MMPs [33], [34]. These alterations in the proteolytic environment may be driven by protease expression both by dermal fibroblasts and the leading edge keratinocytes [35]–[37]. Plasmin has been shown to cleave and thus activate other extracellular proteases important for wound healing [38] and it may thus be speculated that plasmin in this way facilitate wound healing by a fibrinolysis independent manner. Additionally, it has been suggested that other proteases besides plasmin may contribute to fibrin clearance during tissue remodeling. This has been tested by using mice deficient in Plg in addition to specific members of the MMP family, including MMP2, MMP9, or MMP13 [4], [12], [34], [39]. Among these MMP13 deficiency induced a marked delay in wound healing in Plg^−/−^ mice but not to the extent observed when treating Plg^−/−^ mice with the broad spectrum MMP inhibitor Galardin [12], [19], [27]. Thus, several proteases may act in concert to degrade fibrin in the wound matrix. Taking the expression pattern and the multitude of possible functions of plasmin(ogen) into account, the delayed wound healing in Plg^−/−^ mice may very well be directly linked to obstructed cell recruitment or keratinocyte migration. In the current study, we found, that reepithelialized skin wounds in Plg^−/−^ mice still contained large dermal wound gaps, which was significantly larger than that observed in Wt mice. This observation suggests that epidermal wound closure may be less affected by Plg deficiency than reconstruction of the underlying dermal compartment.

Concerning the role of female sex hormones on cutaneous wound healing, it was noted that during the first four weeks post wounding, OVX of Plg^−/−^ mice induced a subtle but statistically significant delay in wound closure compared to sham operated female Plg^−/−^ mice. We speculate, that the enhanced effect of OVX on wound healing in Plg^−/−^ mice compared to Wt mice may relate to specific events during the healing process. Skin wound healing can be divided into several and partially overlapping phases. Following the initial event of coagulation and haemostasis, neutrophils are recruited to the wound site followed by macrophages, which phagocytose remaining neutrophils and secrete tissue growth factors that activate keratinocytes and fibroblasts [40]. Estrogen has previously been shown to modulate the inflammatory response during wound healing through an estrogen dependent down-regulation of the macrophage migration inhibitory factor (MMIF) [27]. The observed delay in the initial phase of wound healing in OVX operated Plg^−/−^ mice in the current study could be related to an additive or synergistic effect of Plg deficiency and depletion of female sex hormones on macrophage migration and activation [41], possibly involving disturbed MIFF activity [27]. As mentioned skin wound healing depends on plasmin driven fibrinolysis but other proteases with fibrinolytic activity seem to take part of the fibrin degradation process in Plg^−/−^ mice [12]. The expression of some of the candidate fibrinolytic proteases e.g. the MT1-MMP [42] have been shown to be regulated by estrogen [43] and could thus be involved in the reduced dependency of Plg in female mice during skin wound healing.

Of notice is that we did not observe any gender dependent difference between sham and OVX operated Wt mice with regard to wound healing. This observation is contradicted by previous reports stating a role for estrogen/estrogen receptor β interaction in cutaneous wound healing [44]. The different outcome of the current study and the one presented by Campbell et al. 2010, may stem from fundamental differences in the model setup (e.g. wound size, age of mice, time between OVX surgery and wounding) or choice of mice strain i.e. FVB/n vs. C57Bl/6 mice, two strains, which in this report has been shown to differ significantly in terms of healing time. Both Wt and Plg^−/−^ C57Bl/6 healed faster than the corresponding FVB mice, thus indicating major differences in either skin composition per se or in one or more of the distinct phases of the wound healing process.

Additional explicatory factors may relate to endogenous differences between male and female mice in skin properties, such as skin strength and mechanical features. In the current study we therefore performed analyses of unchallenged skin biopsies from Wt and Plg deficient male and female mice. In line with other reports, we demonstrated inherent gender differences in normal skin histology. Although these differences were not affected by Plg deficiency, they might affect wound healing in Plg^−/−^ mice. In male mice we observed larger dermal thickness compared to female mice, a parameter that has been reported to be under the influence of male hormones [45]. A direct relationship between dermal thickness and collagen content, and thereby skin strength/mechanical resistance, has been suggested [46]. In relation to skin injury the expression of collagen is markedly increased in fibroblasts in the dermis. This is followed by an extensive remodeling phase, in which the collagen content is degraded by the coordinated action of several collagenolytic proteases, whose expression and activation have been reported to depend on plasmin [47]. Thus, disruption of Plg may have an impact on these processes.

Our data showing that the combined area of fibrin rich lesions in the provisional matrix was significantly lower in Plg^−/−^ female mice compared to Plg^−/−^ male mice could simply be a reflection of the fact that the overall thickness of the skin is smaller in females [45] and that the dermal wound gap in reepithelialized wounds in Plg^−/−^ female mice is smaller than in the corresponding males. However, it is conceivable that the decreased level of fibrin deposition in Plg^−/−^ female mice is secondary to an accelerated fibrinolytic process in comparison with male mice. Interestingly, the differences in dermal fibrin levels between male and female Plg^−/−^ mice did not seem to result in differences within vessel density of the fully healed wounds. However, disregarding gender Plg^−/−^ mice did tend to have fewer CD34 positive vessels underneath the hyperplastic epidermis compared to Wt mice. As extracellular proteases mediate angiogenic processes and that angiogenesis is important for skin wound healing [28]–[30] one of the factors responsible for delayed wound healing in Plg^−/−^ mice may indeed be partly related to hampered angiogenesis.

In conclusion, the presented data show that the effect of Plg deficiency is both gender and tissue specific. The results further suggest that the observed differences in wound healing between male and female Plg^−/−^ mice is not solely related to sex-hormones, though the lack of female sex-hormones, induced by OVX, affected wound healing in female Plg^−/−^ mice to some extent. It is possible that the endogenous differences in skin histology between genders are responsible for the varying effect of Plg deficiency on wound healing, which would explain why the gender effect is restricted to the skin.
